# Regional muscle oxygenation differences in vastus lateralis during different modes of incremental exercise

**DOI:** 10.1186/1476-5918-5-8

**Published:** 2006-07-03

**Authors:** Michael D Kennedy, Mark J Haykowsky, Carol A Boliek, Ben TA Esch, Jessica M Scott, Darren ER Warburton

**Affiliations:** 1 Faculty of Rehabilitation Medicine, University of Alberta, Edmonton, Alberta, Canada; 2 Cardiovascular Physiology and Rehabilitation Laboratory, University of British Columbia, Vancouver, British Columbia, Canada

## Abstract

**Background:**

Near infrared spectroscopy (NIRS) is used to assess muscle oxygenation (MO) within skeletal muscle at rest and during aerobic exercise. Previous investigations have used a single probe placement to measure MO during various forms of exercise. However, regional MO differences have been shown to exist within the same muscle which suggests that different areas of the same muscle may have divergent MO. Thus, the aim of this study was to examine whether regional differences in MO exist within the same muscle during different types of incremental (rest, 25, 50, 75, 100 % of maximum) exercise (1 leg knee extension (KE), 2 leg KE, or cycling).

**Methods:**

Nineteen healthy active males (Mean ± SD: Age 27 ± 4 yrs; VO_2max_: 55 ± 11 mL/kg/min) performed incremental exercise to fatigue using each mode of exercise. NIRS probes were placed on the distal and proximal portion of right leg vastus lateralis (VL). Results were analyzed with a 3-way mixed model ANOVA (probe × intensity × mode).

**Results:**

Differences in MO exist within the VL for each mode of exercise, however these differences were not consistent for each level of intensity. Comparison of MO revealed that the distal region of VL was significantly lower throughout KE exercise (1 leg KE proximal MO – distal MO = 9.9 %; 2 leg KE proximal MO – distal MO = 13 %). In contrast, the difference in MO between proximal and distal regions of VL was smaller in cycling and was not significantly different at heavy workloads (75 and 100 % of maximum).

**Conclusion:**

MO is different within the same muscle and the pattern of the difference will change depending on the mode and intensity of exercise. Future investigations should limit conclusions on MO to the area under assessment as well as the type and intensity of exercise employed.

## Background

Near infrared spectroscopy (NIRS) yields an estimate of muscle oxygenation (MO) patterns in the microcirculation of humans during exercise.[1] NIRS is well suited to the analysis of muscle microcirculation due to the minimal amount of light absorption in arterioles, capillaries and venules compared to feed arteries and veins.[1] NIRS detects changes in light absorption of specific oxylabile chromophores (haemoglobin, myoglobin and cytochrome oxidase) with known absorption spectra in the near-infrared light range (650–1100 nm)[2] Determination of chromophore concentration in biological tissue is based on the modified Beer-Lambert Law,[2] which states that light absorption is due changes in concentration of the aforementioned chromophores. The contribution of myoglobin and cytochrome oxidase to light attenuation is small compared to haemoglobin concentration,[3] and oxygenated haemoglobin absorbs light at 850 nm while deoxygenated haemoglobin absorbs light at 760 nm.[3] Thus, the difference in the amount of light emitted continuously into skeletal muscle at 850 nm and 760 nm compared to the amount absorbed provides an index of oxygenated and deoxygenated haemoglobin concentration expressed as optical density (OD) units.

The findings from the first investigation using NIRS technology during exercise revealed that during maximal intensity exercise, both vastus medialis and vastus lateralis (VL) deoxygenated as measured by the difference in the 760 – 850 nm signal.[4] This finding, has been replicated in dynamic exercise of the knee extensors numerous times [5-16] and is the most common finding for any exercise study utilizing NIRS. Of those investigations which have assessed MO during knee extension (KE) exercise, the most common placement of the NIRS probe is on the distal portion of VL. In addition, all of these investigations have utilized one probe to assess MO where a single probe illuminates an area of approximately 2–6 cm deep [1] by 4–5 cm long.[17] estimated to approximately 16 mL of tissue volume.[18]

Theoretically, microcirculation differences within the same muscle may affect MO despite adequate blood flow to the muscle as a whole.[19] In the animal model one of the causes of regional MO differences is shunting of blood flow to oxidative muscle fibres compared to non-oxidative muscle fibres within the same muscle. [20] Recently, it also has been shown that NIRS measured MO is variable within the same muscle, [21-23]a result confirmed by positron emission tomography (PET).[24] However, these investigations used only discontinuous exercise protocols.[24,21,22] or single leg KE exercise.[21,23,24]

It is recognized that NIRS technology is a tool that allows for a non-invasive exploration of oxygenation in both health and disease.[1] However, with the exception of a few investigations. [21-23] most studies have utilized one probe to measure MO extending MO to the muscle as a whole [3]. Yet this generalization may be erroneous considering that regional MO differences may exist within the same muscle. Thus, the aim of this investigation was to determine whether regional MO differences exist within the same muscle during incremental KE and cycling exercise. We tested the hypothesis that MO values would be different between proximal and distal regions of VL and that this difference in MO would be consistent across exercise intensity and modalities.

## Methods

### Participants

Nineteen participants (Mean ± SD: Age: 27 ± 4 yrs; Ht: 183 ± 6 cm; Wt: 80 ± 7 kg; VO_2_max: 55 ± 11 mL/kg/min; peak power output: 417 ± 80 Watts) performed 3 graded exercise tests to maximum (1 leg KE, 2 leg KE, and cycling). Participants were active healthy males with no history of smoking, heavy drinking and sedentary lifestyle. All participants were recruited from the Vancouver, BC area and provided written informed consent to participate in accordance with guidelines of the Clinical Research Ethics Board (University of British Columbia) and the Health Research Ethics Board (University of Alberta).

### Exercise tests (1 leg KE, 2 leg KE and cycling)

**Figure 1:**
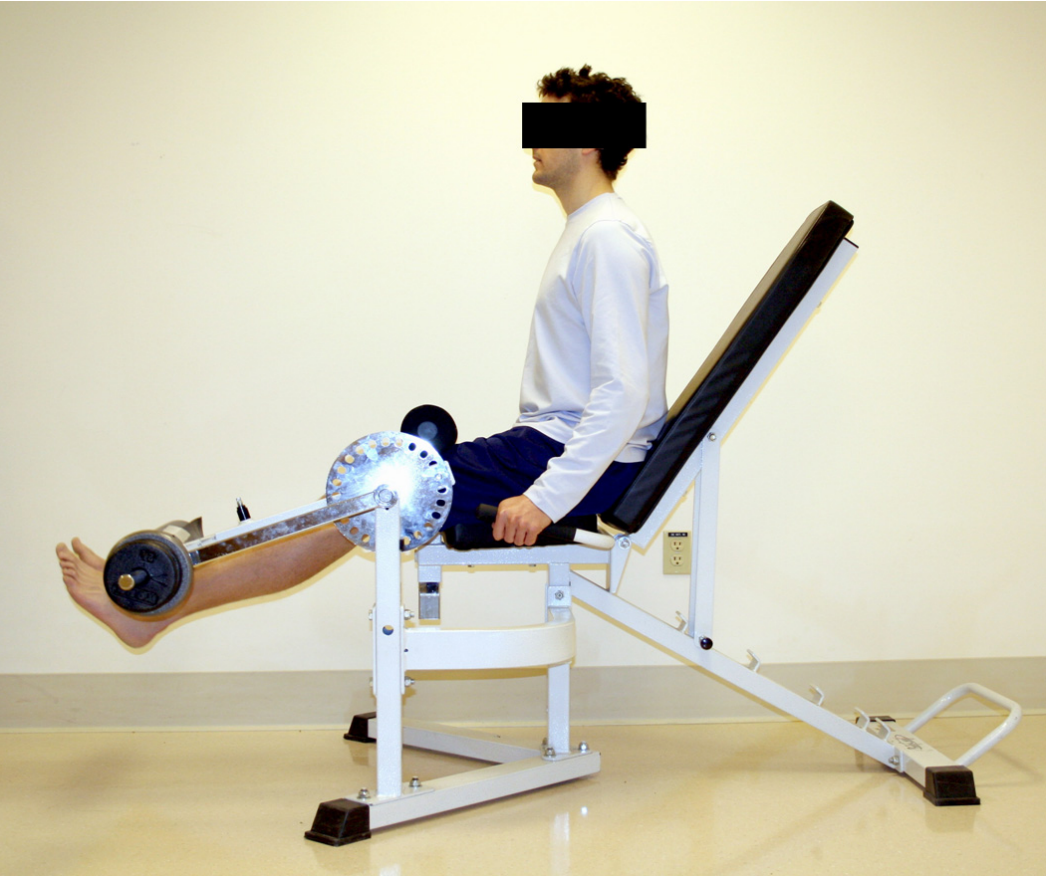
KE machine.

The experimental protocol consisted of these incremental exercise tests to maximum. These tests were administered on 2 different days. On the first day the cycling test occurred followed by a familiarization of the KE apparatus (Figure 1). For familiarization of the KE apparatus, participants were allowed to practice for as long as needed until proper form and timing were achieved. On the second day of testing the 1 leg KE test was performed followed by a break of at least 1 hour, after which the participant performed the 2 leg KE test. The protocols were chosen to minimize any significant decreases in oxygenation which occurs at the onset of exercise due finite oxygen delivery kinetics unequal to metabolic demand [13] Each test is described in more detail below.

#### 1 and 2 leg KE tests

The right leg was used for the 1 leg KE exercise, where the participant's ankle was fastened to the bar of the KE apparatus with tensor bandages. The starting position of the knee was 90° from horizontal, where the participant moved the weight through a range of approximately 80° or full extension. Participants were allowed to hold on to stabilization bars on either side of the seat to reduce any contribution of non-knee extensor muscle activity to the exercise. The bar to which the participant's ankle was attached was adjustable to accommodate the different lower leg lengths of the participants. After rest baseline measures were established, participants exercised for the first minute at a cadence of 40 contractions per minute (cpm) moving a weight of approximately 2.3 kg (4 – 6.5 watts) (i.e. the weight of the KE bar). Each subsequent minute 0.57 kg (~1.5 watts) of weight was added while maintaining a cadence of 40 cpm. The test was stopped when the participant could not consistently maintain a cadence of 40 cpm.

The 2 leg KE exercise occurred a minimum of 1 hour after the 1 KE test, to allow for adequate recovery. For the 2 leg KE test, the same protocol was followed as the 1 leg KE test, except that the weight increments were doubled to 1.14 kg (~3 watts). Both legs were attached to the KE bar with tensor bandages.

#### Cycling test

Participants sat quietly in the cycling position to acquire baseline measures. When resting baseline was established participants cycled at 0 Watts with a slow cadence (less than 50 rpm) for 1 minute. At the start of the 2nd minute an increase to 30 Watts occurred with a cadence of 80 – 85 rpm. An increase of 30 Watts per minute occurred for the rest of the test until volitional exhaustion.

### General design comments

The testing for each participant occurred within a 1 week period. Participants for the duration of the cycling test were able to view both their cadence and power output. Participants for the KE were able to maintain frequency of contraction based on metronome pacing. Strong verbal encouragement was given for the duration of the tests, and music was allowed to further enhance motivation in the tests. A fan was used to reduce any increases in body core temperature which may be seen in a prolonged incremental exercise test. Participants were asked to refrain from heavy exercise up to 48 hours before a test and the importance of proper hydration and energy status was stressed in the information letter.

### Research measures

#### Oxygen uptake, heart rate and SaO2 determination

Oxygen uptake was continuously monitored during all tests using a computerized metabolic measurement cart (Physio-Dyne, Max-1, Fitness Instrument Tech., Farmingdale, NY). Gas analyzers were calibrated with gases of known concentration and the pneumotach (Hans-Rudolph no. 8300, Kansas City, MO) was calibrated with a 3-L syringe before and after each experiment. Heart rate was transmitted and recorded to the metabolic cart wirelessly (Polar Electro Oy, Kempele, Finland). Arterial blood saturation (SaO2) was measured by a pulse oximeter (Ohmeda Biox 3740, Louisville, CO) for every test, with values averaged and recorded every 1 s using a data acquisition system (Powerlab 16/30, ADInstruments, Colorado Springs, CO) and personal computer. A topical vasodilator cream was applied before placement of the oximeter sensor to the pinna of the ear.

#### Muscle oxygenation

Muscle oxygenation was measured with a NIRO 300 (Hamamatsu Photonics, Japan) spatially resolved near infrared oxygenation monitor which measures reflected light at 775, 810, 850, and 905 nm. Measurement of light attenuation at these specific wavelengths allows determination of oxygenated Hemoglobin (ΔHbO2), deoxygenated Hemoglobin (ΔHb), total Hemoglobin (ΔTotHb) and cytochrome oxidase (ΔCtOx).[17,25] In addition, the NIRO 300 provides a derived measure termed the tissue oxygenation index (TOI) (TOI = HbO2/TotHb) providing an index of average saturation of the Haemoglobin volume present within the microvasculature.[1]

**Figure 2:**
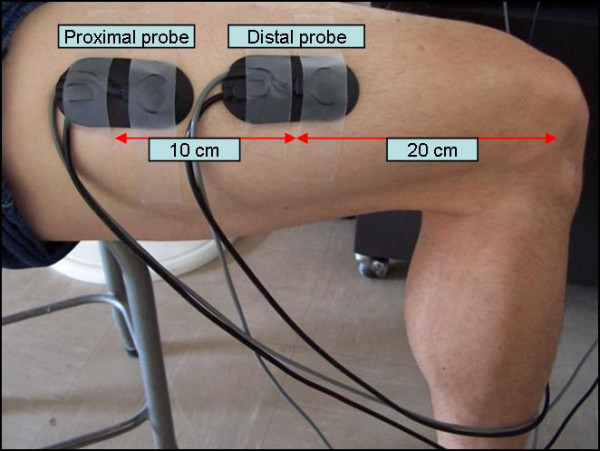
NIRS probes placed on vastus lateralis at both the distal and proximal probe placements.

Muscle oxygenation was evaluated in VL, because VL is utilized in both KE and cycling exercise, although research has shown that the level of recruitment of VL may vary between KE and cycling exercise[26] Despite this limitation, VL is an excellent muscle to evaluate regional MO differences during dynamic exercise, due its size which allows for adequate separation of probes. In addition, placement of probes on VL compared to other knee extensor muscles allows for the least interference in both the KE and cycling movements. The probes were affixed in a black probe holder to ensure maintenance of distance between light source and detection probe. The distal probe was placed in the distal third region of the right leg VL muscle (with the center of the probe approximately 20 cm above the knee joint) (Figure 2). The center of the distal probe was placed approximately 20 cm above the knee joint, to ensure that the probe would remain on a flat portion of the muscle during full extension of the lower limb during knee extension exercise. The center of the proximal probe was placed 10 cm from the center of the distal probe (Figure 2). These placements were made while seated on a chair with the lower leg extended. A small ink mark was placed on the skin to identify the 2 center points for the subsequent test. Both areas were shaved to minimize any influence that hair may have had on light transmission and adipose tissue thickness was measured with Harpenden skinfold calipers at both sites, to ensure that adipose tissue thickness ATT was less than 1.5 cm. The right leg was wrapped with black lycra followed by tensor bandages to affix the probes and eliminate ambient light from contaminating the NIRS signal. The NIRO 300 was calibrated prior to each test and data was collected and saved on-line at a sampling rate of 1 second utilizing a data acquisition system (Powerlab 16/30, ADInstruments, Colorado Springs, CO) and a personal computer.

### Analysis

Measures were averaged every minute for the duration of cycle and knee extensor exercise tests. It was decided that expression of MO values across relative intensity would allow for the best illustration of MO trends from rest to maximum exercise in each mode exercise. Thus, determination of MO values at rest as well as 25, 50, 75 and 100 % of maximum intensity were completed for each participant for each mode of exercise. The rest and exercise data were analyzed with a 3-way repeated measure ANOVA (3 modes of exercise × 2 VL regions × 5 intensities). Post hoc comparisons were considered statistically significant when a mean was not included within the 95 % confidence intervals of its comparison mean. The alpha level was set a priori at p < 0.05.

## Results

MO was different between the distal and proximal regions of VL (proximal = 60.1 ± 10.4 %; distal = 54.5 ± 10.0 %, p < 0.001). There was no difference in adipose tissue thickness at the proximal vs. distal regions of VL. There were significant interactions for VL region (proximal vs. distal) and mode of exercise as well as VL region and intensity of exercise (rest, 25, 50, 75, 100 %). Specifically, in each mode of exercise the proximal MO (1 leg: 60.3 ± 9.1 % vs. 2 leg: 63.4 ± 8.1 % vs. cycling: 56.5 ± 12.3 %) was higher than MO in the distal region of VL (1 leg: 50.4 ± 9.4 % vs. 2 leg: 56.1 ± 9.1 % vs. cycling: 53.5 ± 11.1 %) and this difference was greater for 1 leg and 2 leg KE exercise compared to cycling.

**Figure 3:**
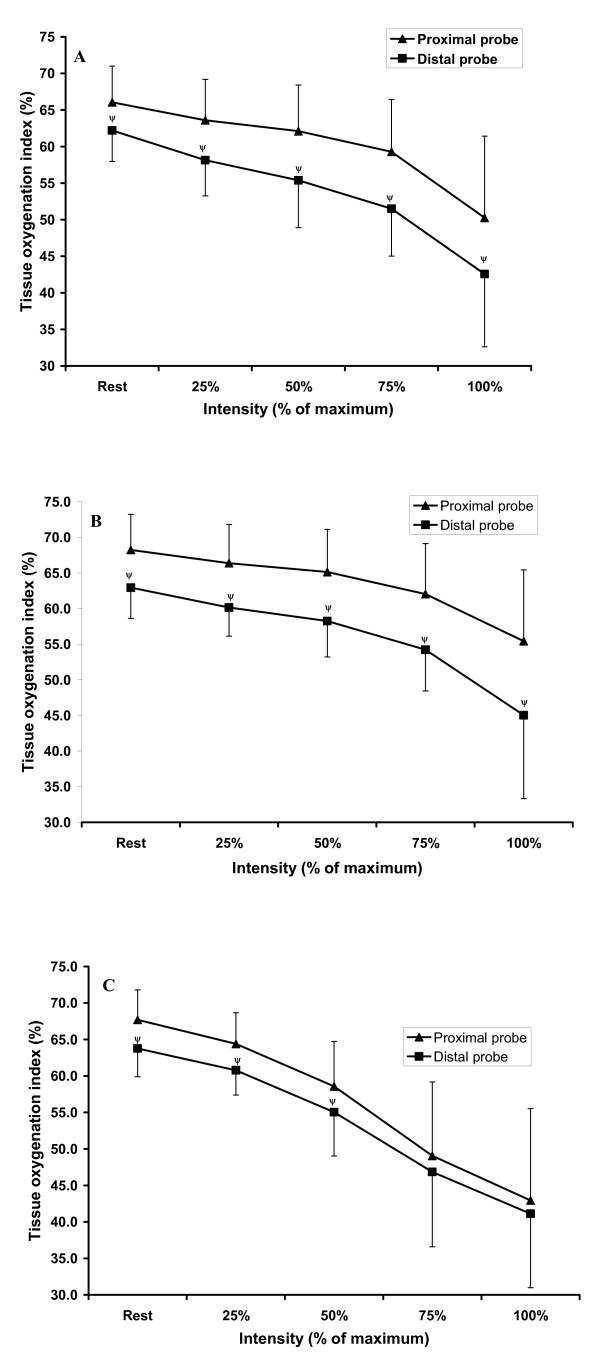
Proximal and distal TOI values for each mode of exercise where, A: 1 leg KE; B: 2 leg KE; C: cycling (ψ, p < 0.05 vs. proximal region of VL).

**Figure 4:**
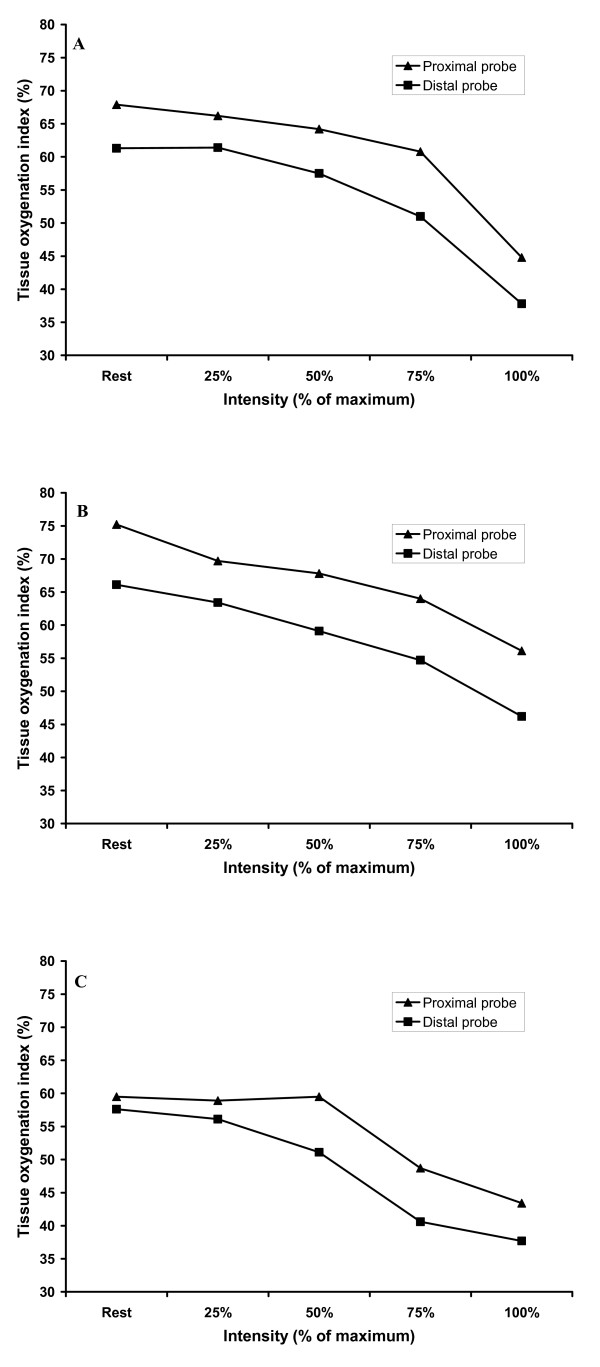
Representative tracing of a single participant for proximal and distal TOI values for each mode of exercise where, A: 1 leg KE; B: 2 leg KE; C: cycling.

There was a significant interaction between VL region (proximal vs. distal), mode of exercise (1 leg KE, 2 leg KE, cycling) and intensity of exercise (rest, 25, 50, 75, 100 %). Further analysis determined that for 1 leg and 2 leg KE exercise MO in the proximal region of VL was higher than the distal MO value at rest and each intensity of exercise (Figure 3A; Figure 3B). However, during cycling the proximal MO value was higher than the distal probe MO at rest, 25 and 50 % (greater than the upper bound of the distal probe 95 % confidence interval) but was not different at 75 and 100 % intensity (Figure 3C). Figure 4A, 4B and 4C provides a representative tracing of a single participant for proximal and distal TOI within each mode of exercise.

## Discussion

The major new finding of this study was that regional MO differences exist within VL during exercise. Specifically, the proximal region of VL was better oxygenated than the distal region of VL and this MO pattern was consistent for all intensities of KE exercise as well as at low intensities of cycling exercise. Others [21,23] have determined that regional MO differences in VL exist, however these investigations only used 1 leg KE exercise to evaluate regional MO differences during either static KE[21] or dynamic KE exercise [23]. Comparatively, the distance between proximal and distal probes was greater in both the Esaki et al [23] and Mizuno et al.[21] investigations compared to the distance between probes in this study. Thus, our findings indicate that significant differences in MO may exist within a smaller area of muscle than has been previously shown.[21,23] In addition, our results reveal that regional differences in MO exist across a full spectrum of intensities in both KE and cycling exercise, a finding which has only been previously found during 1 leg KE exercise.[23]

Comparison of proximal MO compared to distal MO at each workload for 1 and 2 leg KE revealed that the distal region of VL had a lower MO value than the proximal region at rest and throughout exercise (Figure 3A, 3B). The pattern of MO was identical for 1 and 2 leg KE where the difference in MO between regions increased as intensity of exercise increased. This divergence in MO between regions can be attributed to oxygenation in the distal region of muscle decreasing to a greater degree than the MO in the proximal region of VL. These findings support previous research utilizing either static[21] or dynamic [23] 1 leg KE exercise, although differences in methods makes a direct comparison of data difficult.[21,23]

Comparison of proximal versus distal MO during cycling revealed (similar to KE exercise) the distal MO value was significantly smaller than the proximal MO value at rest, as well as at the 25 % and 50 % workloads. However, at 75 % and 100 % intensity there was no difference in MO between regions, caused by a rapid decrease in proximal MO compared to MO change in the distal region of VL. Previously, Kime et al [22] found no difference in MO values at any workload during incremental cycling exercise but did find a decrease in the relative dispersion (range) of values at the heavy workloads. These findings support the idea that relative dispersion of MO decreases due to a functional change in MO from light (< 50 % intensity) to heavy (> 50 % intensity) work in the proximal portion of vastus lateralis. The reasons for this change in MO during the heavy workloads may be due to more uniform recruitment of the entire muscle, [22] or increased perfusion of microvascular units (MVU) relative to metabolic demand in the distal region of VL. In an animal model activation of a single muscle fibre underlying a MVU results in increased flow for that MVU alone [27] and Shinohara et al [28] has shown increased integrated electromyographic activity in vastus lateralis during cycling. Collectively, these findings may support the idea that more uniform recruitment of muscle motor units throughout VL resulted in similar MO values at intense workloads in cycling, although this reasoning remains to be substantiated.

Despite this speculation it is clear that the change in MO values for proximal and distal regions of VL to incremental exercise is different for KE compared to cycling. The factors contributing to a lower distal MO compared to proximal MO for KE are still unclear. Recently Mizuno et al.[21] determined that during isometric KE exercise the electromyographic activity was similar between proximal and distal portions of muscle, thus other factors such as greater intramuscular pressure in the distal portion of muscle, [29] or muscle architecture, [30] may affect regional MO values. It is of interest to note that even at rest there was a significant difference in MO values in all modes of exercise. This would indicate independent of exercise mode, and intensity of exercise that some of the aforementioned ideas contribute to regional MO differences. Further research determining such factors as muscle architecture, blood flow in small vessels of the same muscle and MO may reveal why this difference in MO exists at rest.

## Conclusion

In conclusion, we found that MO was different between proximal and distal regions of VL and this difference in MO values was consistent at all intensities for each mode of exercise except high intensity cycling. These findings support the concept that regional MO differences exist within skeletal muscle both at rest and during exercise. Based on these results, it is suggested that conclusions regarding MO values be limited to the region of muscle under NIRS assessment as well as the type and intensity of exercise employed.

## Competing interests

The author(s) declare that they have no competing interests.

## Authors' contributions

MDK designed the study, coordinated the study, acquired the data, performed the statistical analysis, and drafted the manuscript. MJH participated in the design of the study, interpretation of the results, and preparation of the manuscript. CAB participated in the design of the study, interpretation of the results, and preparation of the manuscript. BTE participated in acquisition of data and coordination of the study. JMS participated in acquisition of data and helped organize the study. DEW participated in coordination of the study, provided the equipment used in the study and provided important revisions to the manuscript. All authors have read and approved the final manuscript.
